# 6-[3-(2,4-Dimethyl­anilino)-2-hydroxy­prop­oxy]-1,8-dihydr­oxy-3-methyl-9,10-dihydro­anthracene-9,10-dione

**DOI:** 10.1107/S1600536809002347

**Published:** 2009-01-23

**Authors:** Xing-Po Wang, Wenfang Xu

**Affiliations:** a School of Chemistry and Chemical Engineering, Shandong University, Shandong 250100, People’s Republic of China; b School of Pharmacy, Shandong University, Shandong 250012, People’s Republic of China

## Abstract

In the title compound, C_26_H_25_NO_6_, the anthraquinone ring system forms a dihedral angle of 15.5 (1)° with the benzene ring of the dimethyl­aniline group. Intra­molecular O—H⋯O hydrogen bonding is observed between the carbonyl and two hydroxyl groups. The mol­ecules are linked into a ribbon-like structure along the [100] direction by O—H⋯N and C—H⋯O hydrogen bonds. The crystal used was twinned *via* a 180° rotation about [100]. The ratio of the two twin components is 0.947 (1):0.053 (1).

## Related literature

For the biological properties of emodin and its derivatives, see: Srinivas *et al.* (2003 ▶); Teich *et al.*, 2004 ▶; Wang & Xu (2005 ▶). For a related structure, see: Wang *et al.* (2006 ▶).
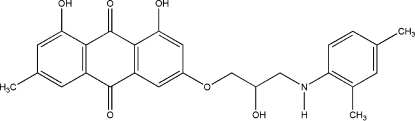


## Experimental

### 

#### Crystal data

C_26_H_25_NO_6_
*M* *_r_* = 447.47
Monoclinic, 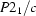

*a* = 5.0668 (3) Å
*b* = 29.7496 (17) Å
*c* = 14.2201 (8) Å
β = 90.530 (4)°
*V* = 2143.4 (2) Å^3^
*Z* = 4
Mo *K*α radiation
μ = 0.10 mm^−1^
*T* = 295 (2) K
0.32 × 0.14 × 0.04 mm

#### Data collection

Bruker APEXII CCD area-detector diffractometer
Absorption correction: multi-scan (*SADABS*; Bruker,2005 ▶) *T*_min_ = 0.969, *T*_max_ = 0.996
17905 measured reflections
4939 independent reflections
2028 reflections with *I* > 2σ(*I*)
*R*_int_ = 0.072

#### Refinement

*R*[*F* ^2^ > 2σ(*F* ^2^)] = 0.072
*wR*(*F* ^2^) = 0.232
*S* = 0.99
4939 reflections
305 parameters
1 restraint
H-atom parameters constrained
Δρ_max_ = 0.65 e Å^−3^
Δρ_min_ = −0.28 e Å^−3^

### 

Data collection: *APEX2* (Bruker, 2005 ▶); cell refinement: *SAINT* (Bruker, 2005 ▶); data reduction: *SAINT*; program(s) used to solve structure: *SIR97* (Altomare *et al.*, 1999 ▶); program(s) used to refine structure: *SHELXL97* (Sheldrick, 2008 ▶); molecular graphics: *SHELXTL* (Sheldrick, 2008 ▶); software used to prepare material for publication: *WinGX* (Farrugia, 1999 ▶).

## Supplementary Material

Crystal structure: contains datablocks I, global. DOI: 10.1107/S1600536809002347/ci2754sup1.cif

Structure factors: contains datablocks I. DOI: 10.1107/S1600536809002347/ci2754Isup2.hkl

Additional supplementary materials:  crystallographic information; 3D view; checkCIF report

## Figures and Tables

**Table 1:** Hydrogen-bond geometry (Å, °)

*D*—H⋯*A*	*D*—H	H⋯*A*	*D*⋯*A*	*D*—H⋯*A*
O1—H1*D*⋯O2	0.82	1.86	2.575 (4)	145
O3—H3*A*⋯O2	0.82	1.83	2.556 (4)	147
O6—H6⋯N1^i^	0.82	2.40	3.218 (5)	177
C15—H15⋯O4^ii^	0.93	2.43	3.334 (4)	164

Symmetry codes: (i) 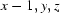; (ii) 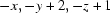.

**Fig. 1.**
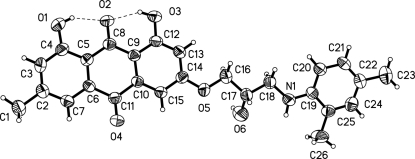
The molecular structure of the title compound, showing the atom-labelling scheme. Displacement ellipsoids are drawn at the 30% probability level. Dashed lines indicate hydrogen-bonding interactions.

**Fig. 2.**
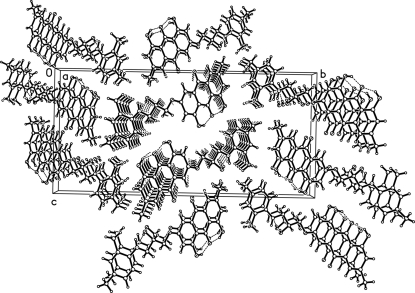
The molecular packing of the title compound, viewed along the a axis. Dashed lines indicate hydrogen-bonding interactions.

## supplementary crystallographic information

### Comment

Emodin and its derivatives have been found to possess diverse biological properties, such as antimicrobial, antiviral, antitumor, anti-inflammatory, anti-oxidant, immunosuppressive, anti-ulcerogenic, fungicidal and chemopreventive activities (Wang & Xu, 2005; Teich *et al.*, 2004; Srinivas *et al.*, 2003). As part of our ongoing research on emodin derivatives (Wang & Xu, 2005; Wang *et al.*, 2006), we report here the crystal structure of the title compound.

The molecular structure of the title compound is illustrated in Fig. 1. The anthraquinone ring system is essentially planar and it forms a dihedral angle of 15.5 (1)° with the benzene ring of the dimethylaniline group. There are two intramolecular O—H···O hydrogen-bonding interactions between the carbonyl and two hydroxy groups (Fig. 1).

The molecules are linked into a ribbon-like structure along the [100] by intermolecular O—H···N and C—H···O hydrogen bonds (Table 1 and Fig.2).

### Experimental

A mixture of emodin (10 mmol) and epichlorohydrin (421 mmol, 33 ml) was stirred under reflux in a solution of potassium hydroxide (10 mmol) in water (3 ml) until the disappearance of the starting material, as evidenced by thin-layer chromatography (about 4 h). After the reaction was over, the solvent was removed *in vacuo* and the residue was partitioned between chloroform (50 ml) and distilled water (20 ml). The organic phase was washed with water (15 ml) and brine (15 ml), and dried over anhydrous sodium sulfate. The solvent was removed to give the key intermediate, 1,8-Dihydroxy-3-methyl-6-(oxiran-2-ylmethoxy)-9,10-dihydroanthracene-9,10-dione (Wang *et al.*, 2006) as a yellow oil, which was purified by flash chromatography (silica gel, petroleum ether–acetone 3:1). To a solution of above intermediate (0.326 g, 1 mmol) in chloroform was added 2,4-dimethylaniline (1.1 mmol). The mixture was refluxed with stirring and monitored by TLC until the reaction was completed. The crude product was purified by column chromatography (petroleum ether–acetone 3:1) to afford the title compound, which was dissolved in methanol (15 ml) and kept at room temperature for 15 d to get yellow single crystals.

### Refinement

All H atoms were placed in geometrically calculated positions and refined using a riding model, with O—H = 0.82 Å, N—H = 0.86 Å and C—H = 0.93–0.98 Å. The *U*_iso_ values were set at 1.2 to 1.5 (hydroxyl and methyl) times the *U*_eq_(carrier atom). The components of the U^ij^ parameters in the direction of the C17—O6 bond were restrained to be equal. The highest residual density peak is located 0.65 Å from atom H17. Attempts to refine this peak as a disordered O6 atom, say O6A, resulted in a very short C17—O6A distance (1.14 Å) and hence the original model was retained. The crystal used was twinned *via* a 180° rotation about the [100]. The ratio of the two twin components is 94.7 (1): 5.3 (1).

### Crystal data

**Table:**

C_26_H_25_NO_6_	*F*(000) = 944
*M**_r_* = 447.47	*D*_x_ = 1.387 Mg m^−^^3^
Monoclinic, *P*2_1_/*c*	Melting point = 469–470 K
Hall symbol: -P 2ybc	Mo *K*α radiation, λ = 0.71073 Å
*a* = 5.0668 (3) Å	Cell parameters from 1559 reflections
*b* = 29.7496 (17) Å	θ = 2.5–19.5°
*c* = 14.2201 (8) Å	µ = 0.10 mm^−^^1^
β = 90.530 (4)°	*T* = 295 K
*V* = 2143.4 (2) Å^3^	Plate, yellow
*Z* = 4	0.32 × 0.14 × 0.04 mm

### Data collection

**Table:**

Bruker APEXII CCD area-detector diffractometer	4939 independent reflections
Radiation source: fine-focus sealed tube	2028 reflections with *I* > 2σ(*I*)
graphite	*R*_int_ = 0.072
φ and ω scans	θ_max_ = 27.6°, θ_min_ = 0.7°
Absorption correction: multi-scan (*SADABS*; Bruker,2005)	*h* = −5→6
*T*_min_ = 0.969, *T*_max_ = 0.996	*k* = −38→30
17905 measured reflections	*l* = −13→18

### Refinement

**Table:**

Refinement on *F*^2^	Primary atom site location: structure-invariant direct methods
Least-squares matrix: full	Secondary atom site location: difference Fourier map
*R*[*F*^2^ > 2σ(*F*^2^)] = 0.072	Hydrogen site location: inferred from neighbouring sites
*wR*(*F*^2^) = 0.232	H-atom parameters constrained
*S* = 0.99	*w* = 1/[σ^2^(*F*_o_^2^) + (0.11*P*)^2^] where *P* = (*F*_o_^2^ + 2*F*_c_^2^)/3
4939 reflections	(Δ/σ)_max_ = 0.001
305 parameters	Δρ_max_ = 0.65 e Å^−^^3^
1 restraint	Δρ_min_ = −0.28 e Å^−^^3^

### Special details

**Table:**

Geometry. All e.s.d.'s (except the e.s.d. in the dihedral angle between two l.s. planes) are estimated using the full covariance matrix. The cell e.s.d.'s are taken into account individually in the estimation of e.s.d.'s in distances, angles and torsion angles; correlations between e.s.d.'s in cell parameters are only used when they are defined by crystal symmetry. An approximate (isotropic) treatment of cell e.s.d.'s is used for estimating e.s.d.'s involving l.s. planes.
Refinement. Refinement of *F*^2^ against ALL reflections. The weighted *R*-factor *wR* and goodness of fit *S* are based on *F*^2^, conventional *R*-factors *R* are based on *F*, with *F* set to zero for negative *F*^2^. The threshold expression of *F*^2^ > σ(*F*^2^) is used only for calculating *R*-factors(gt) *etc*. and is not relevant to the choice of reflections for refinement. *R*-factors based on *F*^2^ are statistically about twice as large as those based on *F*, and *R*- factors based on ALL data will be even larger.

### Fractional atomic coordinates and isotropic or equivalent isotropic displacement parameters (Å^2^)

**Table:**

	*x*	*y*	*z*	*U*_iso_*/*U*_eq_	
C1	−1.0367 (9)	1.13766 (15)	0.5591 (3)	0.0676 (13)	
H2D	−1.1427	1.1638	0.5469	0.101*	
H1B	−0.9129	1.1440	0.6088	0.101*	
H1C	−1.1484	1.1132	0.5774	0.101*	
C2	−0.8902 (8)	1.12513 (13)	0.4719 (3)	0.0524 (10)	
C3	−0.9564 (8)	1.14342 (13)	0.3858 (3)	0.0600 (11)	
H3	−1.0914	1.1645	0.3824	0.072*	
C4	−0.8269 (8)	1.13118 (13)	0.3037 (3)	0.0535 (11)	
C5	−0.6234 (7)	1.09890 (12)	0.3064 (2)	0.0454 (9)	
C6	−0.5564 (7)	1.08053 (12)	0.3954 (2)	0.0431 (9)	
C7	−0.6889 (7)	1.09336 (13)	0.4748 (3)	0.0507 (10)	
H7	−0.6426	1.0804	0.5322	0.061*	
C8	−0.4926 (7)	1.08413 (12)	0.2218 (3)	0.0453 (10)	
C9	−0.2884 (7)	1.05060 (12)	0.2275 (2)	0.0431 (9)	
C10	−0.2116 (7)	1.03164 (12)	0.3150 (2)	0.0409 (9)	
C11	−0.3414 (7)	1.04653 (13)	0.4030 (3)	0.0461 (10)	
C12	−0.1578 (8)	1.03526 (13)	0.1470 (2)	0.0497 (10)	
C13	0.0369 (8)	1.00294 (13)	0.1525 (3)	0.0516 (10)	
H13	0.1215	0.9933	0.0983	0.062*	
C14	0.1057 (7)	0.98486 (12)	0.2389 (3)	0.0457 (9)	
C15	−0.0191 (7)	0.99984 (12)	0.3210 (2)	0.0448 (9)	
H15	0.0298	0.9881	0.3792	0.054*	
C16	0.4139 (8)	0.93266 (13)	0.1716 (3)	0.0549 (11)	
H16A	0.5121	0.9552	0.1370	0.066*	
H16B	0.2815	0.9198	0.1300	0.066*	
C17	0.5967 (8)	0.89666 (13)	0.2081 (3)	0.0526 (10)	
H17	0.7291	0.9111	0.2487	0.063*	
C18	0.7400 (8)	0.87514 (14)	0.1259 (3)	0.0536 (11)	
H18A	0.6129	0.8624	0.0820	0.064*	
H18B	0.8421	0.8977	0.0930	0.064*	
C19	1.0724 (7)	0.81541 (12)	0.1014 (3)	0.0465 (10)	
C20	1.1235 (8)	0.82906 (14)	0.0102 (3)	0.0530 (10)	
H20	1.0373	0.8541	−0.0145	0.064*	
C21	1.3044 (8)	0.80537 (15)	−0.0453 (3)	0.0572 (11)	
H21	1.3358	0.8149	−0.1064	0.069*	
C22	1.4366 (8)	0.76818 (15)	−0.0109 (3)	0.0561 (11)	
C23	1.6400 (9)	0.74387 (17)	−0.0682 (3)	0.0833 (15)	
H23A	1.7896	0.7367	−0.0290	0.125*	
H23B	1.6951	0.7627	−0.1192	0.125*	
H23C	1.5650	0.7166	−0.0929	0.125*	
C24	1.3773 (8)	0.75469 (13)	0.0789 (3)	0.0596 (12)	
H24	1.4617	0.7293	0.1025	0.072*	
C25	1.1997 (8)	0.77663 (13)	0.1365 (3)	0.0521 (10)	
C26	1.1395 (11)	0.76031 (16)	0.2335 (3)	0.0799 (15)	
H26A	1.1720	0.7840	0.2779	0.120*	
H26B	1.2503	0.7351	0.2483	0.120*	
H26C	0.9577	0.7514	0.2364	0.120*	
N1	0.9123 (7)	0.84036 (11)	0.1610 (2)	0.0588 (9)	
H1E	0.9165	0.8349	0.2204	0.071*	
O1	−0.9091 (7)	1.15045 (11)	0.2223 (2)	0.0767 (9)	
H1D	−0.8354	1.1383	0.1779	0.115*	
O2	−0.5598 (6)	1.10083 (9)	0.14266 (17)	0.0608 (8)	
O3	−0.2174 (6)	1.05188 (11)	0.06045 (18)	0.0716 (9)	
H3A	−0.3365	1.0704	0.0648	0.107*	
O4	−0.2717 (6)	1.03226 (10)	0.47874 (18)	0.0662 (9)	
O5	0.2908 (5)	0.95260 (9)	0.25280 (17)	0.0572 (8)	
O6	0.4665 (6)	0.86440 (11)	0.2613 (2)	0.0766 (9)	
H6	0.3221	0.8590	0.2372	0.115*	

### Atomic displacement parameters (Å^2^)

**Table:**

	*U*^11^	*U*^22^	*U*^33^	*U*^12^	*U*^13^	*U*^23^
C1	0.072 (3)	0.069 (3)	0.062 (3)	0.006 (2)	0.010 (2)	−0.009 (2)
C2	0.053 (3)	0.053 (3)	0.051 (3)	0.000 (2)	0.003 (2)	−0.006 (2)
C3	0.065 (3)	0.050 (3)	0.066 (3)	0.016 (2)	0.008 (2)	−0.004 (2)
C4	0.063 (3)	0.045 (2)	0.052 (3)	0.005 (2)	−0.001 (2)	0.0046 (19)
C5	0.047 (2)	0.043 (2)	0.047 (2)	0.0015 (19)	−0.0015 (18)	0.0008 (18)
C6	0.046 (2)	0.046 (2)	0.038 (2)	−0.0011 (18)	−0.0002 (17)	−0.0030 (17)
C7	0.055 (2)	0.056 (3)	0.040 (2)	0.000 (2)	0.0018 (19)	0.0014 (18)
C8	0.051 (2)	0.041 (2)	0.045 (2)	−0.0038 (19)	0.0000 (18)	0.0027 (18)
C9	0.051 (2)	0.038 (2)	0.040 (2)	−0.0008 (18)	0.0012 (18)	0.0020 (17)
C10	0.043 (2)	0.039 (2)	0.041 (2)	−0.0025 (18)	0.0010 (17)	0.0015 (17)
C11	0.044 (2)	0.052 (2)	0.042 (2)	0.0003 (19)	−0.0010 (18)	0.0057 (19)
C12	0.061 (3)	0.050 (2)	0.038 (2)	0.002 (2)	0.0015 (19)	0.0049 (18)
C13	0.060 (3)	0.055 (3)	0.040 (2)	0.004 (2)	0.0061 (19)	−0.0042 (19)
C14	0.049 (2)	0.042 (2)	0.046 (2)	0.002 (2)	−0.0009 (18)	0.0017 (18)
C15	0.050 (2)	0.047 (2)	0.037 (2)	0.0029 (19)	−0.0004 (17)	0.0010 (17)
C16	0.053 (2)	0.056 (3)	0.055 (3)	0.005 (2)	0.006 (2)	−0.006 (2)
C17	0.053 (2)	0.048 (2)	0.057 (2)	0.004 (2)	0.011 (2)	−0.0007 (19)
C18	0.050 (2)	0.057 (3)	0.053 (3)	0.005 (2)	0.001 (2)	−0.0040 (19)
C19	0.049 (2)	0.046 (2)	0.045 (2)	−0.0026 (19)	0.0024 (18)	−0.0039 (18)
C20	0.055 (2)	0.055 (3)	0.049 (2)	0.001 (2)	0.004 (2)	0.0003 (19)
C21	0.063 (3)	0.066 (3)	0.042 (2)	−0.005 (2)	0.011 (2)	−0.006 (2)
C22	0.050 (2)	0.058 (3)	0.060 (3)	−0.002 (2)	0.008 (2)	−0.009 (2)
C23	0.071 (3)	0.081 (4)	0.099 (4)	0.008 (3)	0.020 (3)	−0.015 (3)
C24	0.064 (3)	0.040 (2)	0.074 (3)	0.005 (2)	0.000 (2)	−0.003 (2)
C25	0.062 (3)	0.042 (2)	0.052 (2)	−0.002 (2)	0.000 (2)	0.0045 (19)
C26	0.118 (4)	0.062 (3)	0.059 (3)	0.002 (3)	0.005 (3)	0.014 (2)
N1	0.073 (2)	0.064 (2)	0.0389 (18)	0.015 (2)	0.0049 (17)	−0.0037 (16)
O1	0.094 (2)	0.077 (2)	0.0592 (19)	0.0371 (19)	−0.0034 (18)	0.0122 (17)
O2	0.080 (2)	0.0632 (19)	0.0397 (16)	0.0175 (16)	−0.0012 (14)	0.0092 (13)
O3	0.096 (3)	0.081 (2)	0.0376 (16)	0.0287 (18)	0.0062 (15)	0.0095 (14)
O4	0.078 (2)	0.082 (2)	0.0380 (16)	0.0233 (16)	0.0002 (14)	0.0067 (15)
O5	0.0615 (18)	0.0604 (18)	0.0498 (16)	0.0191 (15)	0.0021 (14)	−0.0034 (13)
O6	0.080 (2)	0.078 (2)	0.072 (2)	−0.0034 (18)	0.0101 (17)	0.0016 (17)

### Geometric parameters (Å, °)

**Table:**

C1—C2	1.498 (5)	C16—H16A	0.97
C1—H2D	0.96	C16—H16B	0.97
C1—H1B	0.96	C17—O6	1.392 (5)
C1—H1C	0.96	C17—C18	1.523 (5)
C2—C3	1.378 (5)	C17—H17	0.98
C2—C7	1.391 (5)	C18—N1	1.440 (5)
C3—C4	1.393 (5)	C18—H18A	0.97
C3—H3	0.93	C18—H18B	0.97
C4—O1	1.353 (4)	C19—C20	1.385 (5)
C4—C5	1.409 (5)	C19—N1	1.393 (5)
C5—C6	1.416 (5)	C19—C25	1.411 (5)
C5—C8	1.448 (5)	C20—C21	1.404 (5)
C6—C7	1.373 (5)	C20—H20	0.93
C6—C11	1.490 (5)	C21—C22	1.380 (6)
C7—H7	0.93	C21—H21	0.93
C8—O2	1.274 (4)	C22—C24	1.375 (5)
C8—C9	1.439 (5)	C22—C23	1.505 (6)
C9—C12	1.403 (5)	C23—H23A	0.96
C9—C10	1.417 (5)	C23—H23B	0.96
C10—C15	1.361 (5)	C23—H23C	0.96
C10—C11	1.487 (5)	C24—C25	1.385 (5)
C11—O4	1.207 (4)	C24—H24	0.93
C12—O3	1.358 (4)	C25—C26	1.496 (5)
C12—C13	1.379 (5)	C26—H26A	0.96
C13—C14	1.384 (5)	C26—H26B	0.96
C13—H13	0.93	C26—H26C	0.96
C14—O5	1.355 (4)	N1—H1E	0.86
C14—C15	1.405 (5)	O1—H1D	0.82
C15—H15	0.93	O3—H3A	0.82
C16—O5	1.445 (4)	O6—H6	0.82
C16—C17	1.505 (5)		
			
C2—C1—H2D	109.5	C17—C16—H16B	110.4
C2—C1—H1B	109.5	H16A—C16—H16B	108.6
H2D—C1—H1B	109.5	O6—C17—C16	112.6 (3)
C2—C1—H1C	109.5	O6—C17—C18	111.0 (3)
H2D—C1—H1C	109.5	C16—C17—C18	109.3 (3)
H1B—C1—H1C	109.5	O6—C17—H17	107.9
C3—C2—C7	117.8 (4)	C16—C17—H17	107.9
C3—C2—C1	121.2 (4)	C18—C17—H17	107.9
C7—C2—C1	120.9 (4)	N1—C18—C17	109.1 (3)
C2—C3—C4	121.9 (4)	N1—C18—H18A	109.9
C2—C3—H3	119.0	C17—C18—H18A	109.9
C4—C3—H3	119.0	N1—C18—H18B	109.9
O1—C4—C3	117.5 (4)	C17—C18—H18B	109.9
O1—C4—C5	122.1 (4)	H18A—C18—H18B	108.3
C3—C4—C5	120.3 (4)	C20—C19—N1	121.9 (4)
C4—C5—C6	117.2 (3)	C20—C19—C25	118.8 (4)
C4—C5—C8	121.6 (3)	N1—C19—C25	119.2 (3)
C6—C5—C8	121.2 (3)	C19—C20—C21	120.5 (4)
C7—C6—C5	120.8 (4)	C19—C20—H20	119.8
C7—C6—C11	119.4 (3)	C21—C20—H20	119.8
C5—C6—C11	119.7 (3)	C22—C21—C20	121.4 (4)
C6—C7—C2	121.9 (4)	C22—C21—H21	119.3
C6—C7—H7	119.1	C20—C21—H21	119.3
C2—C7—H7	119.1	C24—C22—C21	117.0 (4)
O2—C8—C9	120.4 (3)	C24—C22—C23	121.3 (4)
O2—C8—C5	119.7 (3)	C21—C22—C23	121.8 (4)
C9—C8—C5	119.9 (3)	C22—C23—H23A	109.5
C12—C9—C10	117.3 (3)	C22—C23—H23B	109.5
C12—C9—C8	121.6 (3)	H23A—C23—H23B	109.5
C10—C9—C8	121.1 (3)	C22—C23—H23C	109.5
C15—C10—C9	121.5 (3)	H23A—C23—H23C	109.5
C15—C10—C11	118.4 (3)	H23B—C23—H23C	109.5
C9—C10—C11	120.1 (3)	C22—C24—C25	124.0 (4)
O4—C11—C10	121.2 (3)	C22—C24—H24	118.0
O4—C11—C6	120.7 (3)	C25—C24—H24	118.0
C10—C11—C6	118.0 (3)	C24—C25—C19	118.3 (4)
O3—C12—C13	117.2 (3)	C24—C25—C26	122.0 (4)
O3—C12—C9	121.2 (3)	C19—C25—C26	119.7 (4)
C13—C12—C9	121.6 (3)	C25—C26—H26A	109.5
C12—C13—C14	119.7 (3)	C25—C26—H26B	109.5
C12—C13—H13	120.2	H26A—C26—H26B	109.5
C14—C13—H13	120.2	C25—C26—H26C	109.5
O5—C14—C13	125.0 (3)	H26A—C26—H26C	109.5
O5—C14—C15	114.8 (3)	H26B—C26—H26C	109.5
C13—C14—C15	120.2 (3)	C19—N1—C18	121.8 (3)
C10—C15—C14	119.7 (3)	C19—N1—H1E	119.1
C10—C15—H15	120.1	C18—N1—H1E	119.1
C14—C15—H15	120.1	C4—O1—H1D	109.5
O5—C16—C17	106.6 (3)	C12—O3—H3A	109.5
O5—C16—H16A	110.4	C14—O5—C16	118.5 (3)
C17—C16—H16A	110.4	C17—O6—H6	109.5
O5—C16—H16B	110.4		
			
C7—C2—C3—C4	−0.5 (6)	C10—C9—C12—O3	−179.4 (3)
C1—C2—C3—C4	−178.2 (4)	C8—C9—C12—O3	0.7 (6)
C2—C3—C4—O1	178.9 (4)	C10—C9—C12—C13	0.2 (6)
C2—C3—C4—C5	0.7 (6)	C8—C9—C12—C13	−179.6 (3)
O1—C4—C5—C6	−179.1 (4)	O3—C12—C13—C14	180.0 (4)
C3—C4—C5—C6	−0.9 (6)	C9—C12—C13—C14	0.3 (6)
O1—C4—C5—C8	−0.8 (6)	C12—C13—C14—O5	179.1 (3)
C3—C4—C5—C8	177.4 (4)	C12—C13—C14—C15	−1.0 (6)
C4—C5—C6—C7	1.1 (5)	C9—C10—C15—C14	−0.8 (5)
C8—C5—C6—C7	−177.3 (3)	C11—C10—C15—C14	179.5 (3)
C4—C5—C6—C11	−179.5 (3)	O5—C14—C15—C10	−178.8 (3)
C8—C5—C6—C11	2.1 (5)	C13—C14—C15—C10	1.3 (5)
C5—C6—C7—C2	−0.9 (6)	O5—C16—C17—O6	57.8 (4)
C11—C6—C7—C2	179.6 (3)	O5—C16—C17—C18	−178.3 (3)
C3—C2—C7—C6	0.6 (6)	O6—C17—C18—N1	−54.5 (4)
C1—C2—C7—C6	178.3 (4)	C16—C17—C18—N1	−179.3 (3)
C4—C5—C8—O2	0.7 (6)	N1—C19—C20—C21	174.2 (4)
C6—C5—C8—O2	179.0 (3)	C25—C19—C20—C21	−1.8 (6)
C4—C5—C8—C9	−179.1 (3)	C19—C20—C21—C22	−0.2 (6)
C6—C5—C8—C9	−0.8 (5)	C20—C21—C22—C24	1.8 (6)
O2—C8—C9—C12	−0.1 (5)	C20—C21—C22—C23	−177.1 (4)
C5—C8—C9—C12	179.7 (3)	C21—C22—C24—C25	−1.5 (6)
O2—C8—C9—C10	−179.9 (3)	C23—C22—C24—C25	177.5 (4)
C5—C8—C9—C10	−0.1 (5)	C22—C24—C25—C19	−0.5 (6)
C12—C9—C10—C15	0.0 (5)	C22—C24—C25—C26	179.3 (4)
C8—C9—C10—C15	179.9 (3)	C20—C19—C25—C24	2.1 (6)
C12—C9—C10—C11	179.8 (3)	N1—C19—C25—C24	−174.0 (3)
C8—C9—C10—C11	−0.4 (5)	C20—C19—C25—C26	−177.7 (4)
C15—C10—C11—O4	2.9 (5)	N1—C19—C25—C26	6.2 (6)
C9—C10—C11—O4	−176.8 (4)	C20—C19—N1—C18	15.6 (6)
C15—C10—C11—C6	−178.5 (3)	C25—C19—N1—C18	−168.4 (4)
C9—C10—C11—C6	1.7 (5)	C17—C18—N1—C19	−178.8 (3)
C7—C6—C11—O4	−4.6 (6)	C13—C14—O5—C16	−5.0 (5)
C5—C6—C11—O4	176.0 (4)	C15—C14—O5—C16	175.1 (3)
C7—C6—C11—C10	176.9 (3)	C17—C16—O5—C14	−176.9 (3)
C5—C6—C11—C10	−2.6 (5)		

### Hydrogen-bond geometry (Å, °)

**Table:**

*D*—H···*A*	*D*—H	H···*A*	*D*···*A*	*D*—H···*A*
O1—H1D···O2	0.82	1.86	2.575 (4)	145
O3—H3A···O2	0.82	1.83	2.556 (4)	147
O6—H6···N1^i^	0.82	2.40	3.218 (5)	177
C15—H15···O4^ii^	0.93	2.43	3.334 (4)	164

Symmetry codes: (i) *x*−1, *y*, *z*; (ii) −*x*, −*y*+2, −*z*+1.
